# Supplementary material for: Effect of artificial lung fiber bundle geometric design on micro‐ and macro‐scale clot formation
Source: Bioeng Transl Med. 2024 Jul 16;9(6):e10699. doi: 10.1002/btm2.10699 (PMC11558184; doi:10.1002/btm2.10699)
Supplement: Supplementary file 1 — Appendix S1: Supporting information. [file BTM2-9-e10699-s001.docx]

**Supporting Information**

Supplementary Table 1.

| Device Name | Path Length | Surface Area | Blood Flow |
| --- | --- | --- | --- |
| FB-F | 3.12 inch | 0.65 m2 | 3.5 L/min |
| APL (ambulatory pump lung) | .85 inch | .8 m2 | 3.5 L/min |
| cTAL | 1.49 inch | 2.4 m2 |  |
| Sorin Inspire PHISIO | | 1.75 m2 | 6-8 LPM |
| Maquet Quadrox | | 1.8 m^2^ | 0.5-5 LPM |

Supplementary Table 1:

Hollow fiber bundle dimensions of commercial and pre-clinical artificial lungs.

Supplementary Information 2.

//Open image stack you are interested in that is in binary. Make sure it is saved as a Tiff stack
//that has already been cropped and aligned using the 3DSlicer software
//
//This macro runs through the slices of an already opened TIFF stack to collect the histogram
//value for each bin, 0-255, a grayscale 8-bit image
 
run("Clear Results");
setOption("ShowRowNumbers", false);
for (slice=1; slice<=nSlices; slice++) {

“setSlice(slice)”; //sets the slice as the current slice
getRawStatistics(n, mean, min, max, std, hist);
for (i=0; i<hist.length; i++) {
setResult("Value", i, i);
setResult("Count"+slice, i, hist[i]);
}
}
